# Preliminary results of phase I trial of oral uracil/tegafur (UFT), leucovorin plus irinotecan and radiation therapy for patients with locally recurrent rectal cancer

**DOI:** 10.1186/1477-7819-4-83

**Published:** 2006-11-22

**Authors:** Masayoshi Yasui, Masataka Ikeda, Mitsugu Sekimoto, Hirofumi Yamamoto, Ichiro Takemasa, Takafumi Ueda, Junzo Shimizu, Mutsumi Fukunaga, Osamu Suzuki, Takehiro Inoue, Morito Monden

**Affiliations:** 1 Department of Surgery, Graduate School of Medicine, Osaka University, Osaka, Japan; 2 Department of Orthopeadics, Graduate School of Medicine, Osaka University, Osaka, Japan; 3 Department of Surgery, Sakai Municipal Hospital, Osaka, Japan; 4 Department of Radiation Oncology, Graduate School of Medicine, Osaka University, Osaka, Japan

## Abstract

**Background:**

Surgical attempts for locally recurrent rectal cancer often fail due to local re-recurrence and distant metastasis. Preoperative chemoradiation may enhance better local control and survival. The aim of this study was to assess the safety of oral uracil and tegafur (UFT) plus leucovorin (LV), and irinotecan combined with radiation and determine the maximum-tolerated dose (MTD) and dose limiting toxicity (DLT) of the triple drug regimen.

**Patients and methods:**

Patients with locally recurrent rectal cancer received escalating doses of irinotecan on days 1, 8, 15, and 22 (starting at 30 mg/m^2^, with 10 mg increments between consecutive cohorts) and fixed doses of UFT (300 mg/m^2^) plus LV (75 mg/day) on days 3 to 7, 10 to 14, 17 to 21, and 24 to 28. Radiation was given 5 days per week totaling 40 to 50 Gy (2Gy/day).

**Results:**

Six patients were treated at the starting dose, and 2 received the full scheduled chemoradiotherapy. The other 4 patients had grade 3 diarrhea and diarrhea was the DLT. One patient had partial response and he had subsequently radical surgical resection. Median progression free survival for local recurrence was 320 days.

**Conclusion:**

Irinotecan plus UFT/LV with concomitant radiotherapy in patients with locally recurrent rectal cancer was not feasible due to diarrhea in this setting. Modification of the treatment is needed.

## Background

Local recurrence of rectal cancer is a formidable problem after surgery for primary advanced rectal cancer. It has been reported to be 5% to 30% after curative resection [1,2]. The prognosis for these patients is usually dismal in terms of survival and quality of life. Twenty to 50 % of these patients had local recurrence in the absence of distant metastasis [1,3]. In such patients, surgical intervention is the best treatment choice for cure, however, extended radical surgery including sacrectomy is often required to obtain negative surgical margin [4-8]. In spite of these aggressive attempts, the incidence of local re-recurrence is still very high ranging from 30% to 70% [5,6,9-11]. Multidisciplinary treatment approach is necessary for better outcome.

In order to reduce local failure after surgery for primary advanced rectal cancer, Heald has introduced total mesorectal excision (TME) [12]. The Dutch trial was designed to evaluate the benefit of neoadjuvant therapy in addition to TME. Subgroup analysis in the trial, preoperative radiotherapy could not improve local recurrence rate in patients with a positive circumferential margin or in patients requiring abdominoperineal resection [13]. German study group demonstrated the usefulness of preoperative chemoradiotherapy for locally advanced rectal cancer to reduce local failure [14]. Gerard *et al*., [15] (Fédération Française de Cancérologie 9203 trial) and Bosset *et al*., [16] (EORTC 22921 trial) have also reported decreased local failure rate by the addition of concurrent chemotherapy in a neoadjuvant setting in comparison with radiation only treatment. Many U.S. randomized trials have indicated survival benefits for both chemotherapy and concurrent chemoradiotherapy in the treatment of rectal cancer [17,18]. Adding other chemotherapeutic agents, which has been proven to improve the outcome in advanced/metastatic disease, might potentially add to an already proven curative benefit. There are a number of new agents that have been developed for the treatment of patients with colorectal cancer. Many trials with new agents for concurrent radiotherapy are ongoing [19-23]. Several authors reported pilot studies of newer agents, including capecitabine, UFT, oxaliplatin, irinotecan combined with 5-FU and radiotherapy in the treatment of rectal cancer. Mohiuddin *et al*., [24] recently published phase II trial which indicated irinotecan and 5-FU in combination with radiotherapy have no better pathological complete response (pCR) rate than infusional 5-FU and radiotherapy as preoperative treatment in rectal cancer. However, Mitchell et al reported good results in pCR rate of preoperative chemoradiation therapy with irinotecan and 5-FU [25].

Several trials regarding preoperative radiotherapy or chemoradiotherapy for locally recurrent rectal cancer has been reported as feasible and effective for improved respectability [26-29]. Most of these studies, main chemotherapeutic agents are 5-fluorouracil (5-FU), mitomycin C, and cisplatin. As complete pathologic response to preoperative therapy was predictive for an improved survival, [29,30] and patients with locally recurrent rectal cancer sometimes develop and die of distant metastasis, administration of effective and sufficient doses of active agents concomitant with radiotherapy might improve survival. Irinotecan has been shown to be effective in 5-FU refractory patients and irinotecan combined with 5FU showed good results in metastatic and recurrent colorectal cancer. Adding irinotecan to 5-FU with concomitant radiotherapy may improve already proven curative effect of 5FU based chemoradiation therapy of the local recurrent rectal cancer.

Irinotecan, oral tegafur/uracil (UFT), and leucovorin (LV) might be expected to improve the local tumor regression of radiotherapy without infusional 5-FU. Combined-modality therapy with triple drugs and radiotherapy for the local recurrence of rectal cancer has not been reported. In the present study, we initiated a phase I trial to establish a combination of triple drugs with concomitant radiotherapy for locally recurrent rectal cancer.

## Patients and methods

### Eligibility and evaluation

This phase I trial was two-institutional prospective study. Patients with locally recurrent rectal cancer were eligible for entry onto this study. Additional inclusion criteria were as follows: age 20–75 years, World Health Organization (WHO) performance status of 0–1, adequate marrow (12,000 ≥ leukocyte count ≥ 4,000/μL, platelet count ≥ 100,000/μL), liver (AST/ALT < 100 U/L, total-bilirubin ≤ 1.5 mg/dl), and renal (serum creatinin ≤ 1.5 mg/dl) function, and ability of oral intake. All patients provided written informed consent. The protocol was reviewed and approved by the institutional review board and the study was performed according to the Declaration of Helsinki. Patients were excluded if they have uncontrolled heart failure, previous history of radiotherapy, prior malignant disease, chronic diarrhea, and pregnancy or lactation. Pretreatment disease evaluation included physical examination, digital rectal examination, and sigmoidoscopy, computed tomography of chest, abdomen, and pelvis, and positron emission tomography in possible cases.

### Study design and treatment plan

The primary objective of this study was to determine recommended irinotecan dose when combined with UFT, LV and radiation therapy in patients with locally recurrent rectal cancer by monitoring the dose-limiting toxicities (DLTs) at each dose level. Secondary object of this study was efficacy and overall survival.

### Chemotherapy

**Figure 1:**
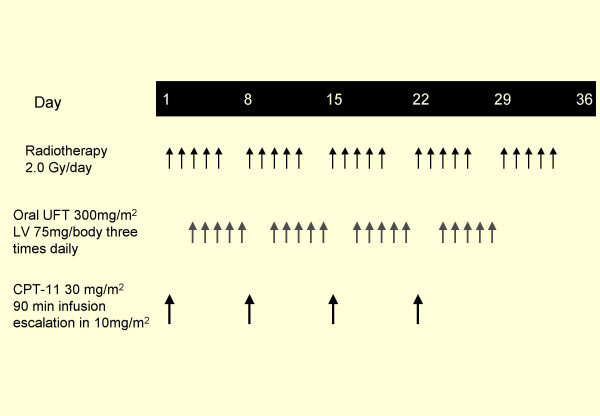
Schema of treatment regimen.

The treatment consists of escalating doses of irinotecan as a 90-minites infusion on day 1 of radiotherapy (RT) for 4 consecutive weeks (days 1,8,15 and 22). Patients received UFT/LV orally 3 times a day (every 8 hours) for the days 3–7, 10–14, 17–21, 24–28, q5w. The dose of UFT and LV were fixed at 300 mg/m^2^/day and 75 mg/body/day, respectively, and starting dose of irinotecan was 30 mg/m^2 ^(Figure 1). This dose represented 30% of the dose intensity achieved with the recommended dose of 100 mg/m^2^, and the dose was escalated of 10 mg/m^2 ^in subsequent groups of patients up to 70 mg/m^2 ^to determine maximum tolerated dose (MTD). The doses of UFT and LV were recommended dose of these two-drug combination therapy. Three patients were initially enrolled at each dose level. If none of these three experienced DLT (defined later), then the next three patients were enrolled at the next higher level. If one patient experienced DLT, then three more patients were recruited at the same level. If no more than one of six patients had DLT, then the next cohort of patients was treated at the next level. If two or more patients had DLT, then that level was considered to MTD, and the level immediately preceding that level was designated as the recommended dose (RD). Further five patients were then enrolled at the RD to investigate the toxicities and tolerability of that level. Patients were evaluated for toxicity weekly while on study, and all toxicities were graded using the National Cancer Institute Common Toxicity Criteria version 3.0. To avoid any bias relating to GI adverse events, antiemetic prophylaxis were administrated to all patients

If a patient reported one of the following adverse events on the day of planned irinotecan administration, chemotherapy was to be interrupted until adverse effect resolved : leukopenia ≥ grade 2, neutropenia ≥ grade 2, platelets ≤ 100000/μL, diarrhea ≥ grade 1, and other adverse events ≥ grade 3 (excluding alopecia, nausea, vomiting, fatigue, and appetite loss). UFT/LV administration was withheld in the event of grade 3 or 4 leukopenia and neutropenia, platelets ≤ 100000/μL, diarrhea ≥ grade 1, AST/ALT ≥ grade 2, stomatitis ≥ grade 2, and other adverse events ≥ grade 3 (excluding alopecia, nausea, vomiting, fatigue, and appetite loss).

### Radiotherapy

RT was delivered with a linear accelerator with using 10-MV photons with anterior-posterior opposing field or three-field technique consisting of a posterior and two lateral fields. For three-dimensional treatment planning purpose, all patients had a computed tomographic (CT) scan in the treatment position. A planned irradiation was given in daily fractions of 2.0 Gy, 5 days a week for 5 consecutive weeks, resulting total dose of 40 to 50 Gy. The clinical target volume (CTV) included the recurrent tumor with 1–1.5 cm margin, sacrum, and the presacral space. The upper border of the CTV was at the L5-S1 interspace. The lower field border was 3 cm below the macroscopic tumor. Planning target volume (PTV) margin was 5 mm from the CTV. Unless a patient had a following adverse events: leukopenia ≥ grade3, neutropenia ≥ grade3, thrombocytopenia ≥ grade3, diarrhea ≥ grade 2, the irradiation was not interrupted. In these occurrences, RT was withheld until adverse events resolved.

### Dose limiting toxicities

DLT was defined by the occurrence of one of the following adverse events: 1) grade 4 hematologic toxicity, 2) any grade 3 non-hematologic toxicity except for nausea and vomiting. 3) RT cessation of ≥ one week, 4) cancellation of irinotecan administration ≥ twice.

All the patients have been carefully followed by the radiation oncologist and the surgeon. The follow-up ranges from 650 to 721 days with a median follow-up of 697 days. Two patients had died and were followed until death. Objective tumor response of locally recurrent mass was assessed according to World Health Organization criteria. Briefly, complete response (CR) was defined as the complete disappearance of all recurrent tumors, partial response (PR) as ≥ 50% reduction by the product of the longest cross-diameters, stable disease (SD) as < 25% reduction or increase and progressive disease (PD) as ≥ 25% increase. Tumor size was determined by CT-PET fusion images utilizing Standard Uptake Values [31]. The status of distant metastases was not considered in this study. In case of patients with surgical intervention, confirmation of a 4-week duration was not applied.

## Results

Six patients were enrolled onto this two-institutional trial between January 2004 and May 2004. Patient basic characteristics are listed in Table 1

**Table 1:** Patient characteristics in 6 patients.

**Case**	**Age**	**Sex**	**Time to LR **(months)	**Initial surgery**	**Recurrent site**	**ECOG PS**
#1	54	Male	9	AR	Local	0
#2	55	Male	72	LAR	Local	0
#3	66	Male	65	AR	Local, lung	0
#4	62	Male	11	TPE	Local, lung	0
#5	50	Male	22	LAR	Local, lung	0
#6	56	Male	23	LAR	Local, liver, LN	0

LR, local recurrence; ECOG, Eastern Cooperative Oncology Group; PS, performance status; AR, anterior resection; LAR, low anterior resection; TPE, total pelvic exenteration

### Toxicities

In the first enrollment, 3 patients were treated at level 1 between January and February 2004. Case 3, 66-year-old-male, treatment had to be interrupted at day 18 because of grade 3 diarrhea (with grade 1 abdominal pain). The patient recovered within 1 week, so the rest of chemotherapy was resumed. The other 2 patients did not have any DLTs. We recruited then an additional three patients at the same dose level. In these patients, all 3 patients had DLTs. In one patient (case 4), therapy had to be discontinued due to grade 3 diarrhea at day25 and bowel fistula requiring surgical intervention. In another patient (case 5), he had treatment interruption because of reversible grade 3 diarrhea (at day21) lasting for less than 7 days. The other patient (case 6) had grade 3 diarrhea at day19 as well.

Of six patients treated on dose level I, four patients had DLTs, including 4 patients with grade 3 diarrhea, and one patient with bowel fistula which required surgical intervention. Consequently dose escalation stopped at a dose of 30 mg/m^2^, and diarrhea was considered as a DLT. All adverse events are listed separately for the six patients in Table 2.

**Table 2:** Maximum severity of adverse events in each 6 patients.

NCI-CTC grade							
Case#							

		#1	#2	#3	#4	#5	#6

Hematological							
	Leukopenia	0	0	0	3	0	2
	Neutropenia	0	0	1	2	0	2
	Anemia	1	1	1	2	1	0
	Thrombocytopenia	0	0	0	0	0	0
Gastrointestinal							
	Appetite loss	1	1	1	2	1	0
	Diarrhea	0	1	3	3	3	3
	Nausea/vomiting	1	1	0	2	1	0
	Abdominal pain	1	1	1	3	1	2

Grade 3 diarrhea occurred at day 18 (#3), 25 (#4), 21 (#5), and 19 (#6)

Case #4 had bowel fistula which required surgical intervention.

Grade 2 or more hematological adverse events were observed in 2 patients. One patient developed grade 3 leukopenia, and one patients developed grade 2 leukopenia lasting for less than 7 days, both of whom also developed grade 2 neutropenia. Grade 2 anemia was observed in one case. No blood transfusions were required during chemoradiotherapy. One patients who had bowel fistula developed grade 3 abdominal pain and grade 2 appetite loss and nausea/vomiting.

The median percentage of given dose per planned chemotherapy of irinotecan and UFT delivered to patients was 100 % (mean; 95.8%) and 96.7 % (mean; 92.5%), respectively.

### Tumor response and survival

The response rate of the locally recurrent tumor was 17%. There were one PR patient, 4 SD patients and one PD patient. The patient with PR could proceed to surgical resection of locally recurrent tumor with curative intent. With a median follow-up of 697 days, two patients died. Median progression free survival time of locally recurrent tumor for 5 patients without operation was 320 days. Median overall survival time has not been reached yet.

## Discussion

There is no standard therapy for the treatment of locally recurrent rectal cancer, especially for tumors such as those fixed to the pelvic walls. External beam radiotherapy, intraoperative radiotherapy, chemotherapy, and surgery or combination of these modalities has been employed. These treatments gave certain results with 5-year survival between 20% to 50%, however systematic treatment strategy still remains to be established. Patients with locally recurrent rectal cancer have been routinely included in published phase I and II trials of chemoradiation therapy, and curative outcome in these patients has been previously reported. Mohiuddin *et al*., reported the prognostic significance of post chemoradiation pathologic stage following preoperative chemoradiation for recurrent rectal cancer patient who were treated with bolus/continuous 5FU combined with radiation therapy [32]. In the treatment of metastatic colorectal cancer, irinotecan has been shown to be effective, so we hypothesize preoperative irinotecan, UFT and concurrent radiotherapy might improve tumor regression and potentially have survival benefits compared to currently available chemoradiation regimen in the rectal cancer patients with local failure.

Firstly, we have tried to determine recommended dose of irinotecan with concomitant radiotherapy, when UFT and LV doses were fixed. Irinotecan is a promising chemotherapy agent and is also a new class of chemotherapeutic radiation sensitizer [25,33]. We did not administer UFT and CPT-11 simultaneously because simultaneous administration of 5-FU and CPT-11 might have the small chance of severe GI toxicity. Shimada *et al*., reported unexpected mild toxicity in the combined CPT-11 and 5-FU regimen [34]. Sasaki *et al*., reported pharmacokinetic interaction of 5FU and CPT-11 which showed the plasma concentration or AUC of CPT-11 was higher in the combined CPT-11 and 5FU regimen than in the CPT-11 alone regimen [35].

We have reported that separate administration of CPT-11 and UFT in this regimen did not alter the pharmacokinetics of SN-38, a major active metabolite of CPT-11 [36]. As we considered CPT-11 as the key drug to enhance the effect of radiation in this regimen, we gave CPT-11 on the beginning day of radiation therapy. Therefore, UFT was not administered concurrently on radiotherapy days in this study.

A total of 6 patients were entered onto the study. On dose level I, 4 of 6 patients developed grade 3 diarrhea, which was accompanied by grade 3 leukopenia and abdominal pain in one patient. In this patient, bowel fistula had occurred and subsequently surgical intervention was required. As we encountered DLT on dose level I, we have discussed about whether we should recruit patients on dose level 0, irinotecan 20 mg/m^2^. As irinotecan 20 mg/m^2^, one fifth dose of recommended dose of monotherapy in Japan, might be too low to have biological activity for metastatic region outside the radiation fields, we have chosen to use different chemotherapy schedule to examine the recommended dose of irinotecan with UFT/LV in combination with radiotherapy. As grade 3 diarrhea occurred around the 3^rd ^week, we modified the treatment schedule to put chemotherapy-rest during the third week, and oral UFT/LV were to be administered on the same day of radiation to make complete chemoradiatherapy-free days. We have reached DLT at a low dose of irinotecan. Japanese patients are susceptible to have irinotecan induced GI toxicity, especially diarrhea. Maximum approved weekly dose of irinotecan alone in Japan is 100 mg/m^2 ^which is much lower than European/American dose [37].

In the present study, treatment response of 6 patients was not favorable according to the WHO criteria. However, in one patient, surgical resection was performed 4 weeks after this chemoradiotherapy because of prominent tumor regression, otherwise he was inoperable. This patient underwent super low anterior resection with high sacral bone resection for curative intent.

5-FU, leucovorin, cisplatin, and mitomycin C have been used in combination with radiotherapy for the purpose of radio-sensitizer. Their regimens were feasible and no severe toxicities were reported [26,27,37,38]. UFT plus leucovorin and radiation have been extensively investigated in both primary advanced or recurrent rectal cancer [39-41]. DLT was diarrhea as in this study, and recommended dose for the concomitant radiation was 350 mg/m^2^/day UFT + 90 mg LV [39,40]. In the recent years, capecitabine, irinotecan, and oxaliplatin have been used for primary advanced or inoperable rectal cancer [42-44]. Mohiuddin *et al*., reported randomized phase II trial which showed that combining irinotecan (50 mg/m^2^) and 5-FU with radiotherapy was tolerable and efficacious [24]. In contrast to the study, we have reached DLT at a low dose of irinotecan (30 mg/m^2^). Japanese patients are susceptible to have irinotecan induced GI toxicity, especially diarrhea. Maximum approved weekly dose of irinotecan alone in Japan is 100 mg/m^2 ^which is much lower than European/American dose [45]. These combined modality have achieved clear resection margins and tumor "downstaging", and thus may improve long-term local control and survival rate [27,28,37,43]. However, combined-modality therapy of the triple drugs with radiotherapy for the local recurrence of rectal cancer has not been reported. A progress in our regimen might have high remission rate and may establish a chemoradiotherapy as a preoperative treatment for the patients with locally recurrent rectal cancer.

## Summary

This study was not able to demonstrate the feasibility of irinotecan, UFT/LV with concomitant radiotherapy in patients with locally recurrent rectal cancer due to diarrhea. We are now investigating this triple-drug regimen with modified treatment schedule. Further insight into treatment schedule and patient management after surgery may establish a safer and effective treatment for the locally recurrent rectal cancer.

## Competing interests

The author(s) declare that they have no competing interests.

## Authors' contributions

**MY **have made substantial contributions for data acquisition, patient treatment, and drafting the manuscript. **MI **participated in the concept and design of the study, and have revised the manuscript. **MS, HY, IT, JS**, and **MF **participated in the concept and design of the study and patient treatment substantially. **TU **participated in the concept of this study as an orthopedist, and also participated in the patient treatment. **OS **and **TI **participated in the concept and design of this study as radiation oncologist. They have also treated patients. **MM **have given final approval of the version to be published.

All authors read and approved the final manuscript.
